# Serial crystallography using synchrotron radiation

**DOI:** 10.1107/S2052252514000499

**Published:** 2014-02-28

**Authors:** Michael G. Rossmann

**Affiliations:** a Department of Biological Sciences, Purdue University, West Lafayette, IN 47907, USA

**Keywords:** protein microcrystallography, serial crystallography, **in vivo**-grown microcrystals

## Abstract

A brief history is given of how X-ray diffraction data from crystals have been recorded. Today there are new possibilities, spawned by the availability of free electron lasers that produce powerful femtosecond long X-ray pulses.

The first X-ray diffraction images were of a stationary CuSO_4_.5H_2_O crystal, recorded on photographic film with white X-rays, now called Laue photography in honor of Max von Laue’s discovery in 1912 (Ewald, 1952 ▶). Although W. L. Bragg replaced film by an ionization chamber that could select a single reflection at a time, photographic film was re-introduced as a detector with the advent of the rotation camera and filtered monochromated radiation. The subsequent Weissenberg improvement of the rotation camera introduced screens to block out all but one layer of reciprocal space (Weissenberg, 1924 ▶). All reflections recorded on one Weissenberg image were of reflections that had completely passed through the surface of the Ewald sphere and were therefore ‘full’ reflections. Their relative intensities could be measured by visual comparison with a prepared scale of increasing intensities, reflection by reflection. Until the introduction of the first electronic computers in the mid 1950s, most computations were limited to two-dimensional analyses. Thus, collecting the data for a single central reciprocal lattice plane on a Weissenberg camera was generally all that was needed for a structure determination. In the 1940s, Martin Buerger (1944 ▶) introduced a further simplification with the invention of a precession camera that gave images of full reflections presented as undistorted views of reciprocal space, making indexing certain and simple. But these simplifications, together with the introduction of film scanning devices as well as primitive electronic computers, made it possible to contemplate systematic three-dimensional diffraction data collection. However, that required scaling the intensity measurements of reflections on individual planes of reciprocal space onto a common scale, usually by means of simple least-squares procedures (Hamilton *et al.*, 1965 ▶).

In the 1970s, Arndt & Wonacott (1977 ▶) observed that introducing layer line screens for Weissenberg and precession photography was an enormous waste of crystal life, as the vast majority of reflections were being stopped by the layer line screens without being recorded. Thus, Arndt re-introduced oscillation photography. This had been used earlier for quite a few decades, but indexing of reflections was uncertain, which was another reason why precession photography became popular. However, precision image scanning had become possible permitting the accurate indexing of a crystal whose orientation was accurately set experimentally (Rossmann, 1979 ▶). There was, however, another problem: the reflections that occurred at the start or end of the oscillation range were only partially recorded. Initially, the procedure was merely to throw away these partial reflections and use only the remaining full reflections for scaling images and intensity measurements. However, that became very wasteful when the unit cell sizes were large (as for virus crystals), requiring small oscillation angles to avoid excessive overlapping of reflections and thereby decreasing the proportion of fully recorded reflections. This problem was largely solved by correcting the partial reflection intensities (*I*_obs_) with calculations of their partialities (*p*), which then gave the estimate of the full intensity (*I*_obs_/*p*). However, calculation of the partiality required an accurate knowledge of the exact orientation of each crystal in the data set, the cell dimensions, and the effective mosaic spread. These calculations also required a scaled data set in a procedure that has become known as ‘post refinement’ (Winkler *et al.*, 1979 ▶; Rossmann *et al.*, 1979 ▶; Kabsch, 2010 ▶). The scaling was performed with only the available full reflections, but the final complete data included all the recorded diffraction data corrected for partiality.

The next major advance was the introduction of freezing crystals which gave a much longer life to the radiation sensitive crystals (Haas & Rossmann, 1970 ▶; Hope *et al.*, 1989 ▶). That made it possible to obtain a complete three-dimensional data set and made it possible to add the partially recorded reflections on sequential images to determine the intensity of full reflection. The latter was especially important because freezing crystals usually significantly increased the mosaic spread to where the mosaic spread was greater than the oscillation angle resulting in no full reflections that could be used for scaling. Although many authors, including the authors of the paper discussed here (Gati *et al.*, 2014 ▶), seem to consider merging data from different crystals a disadvantage, it is probably an advantage. That is because different crystals have different shapes and different vitreous ice environments. Thus, merging data from different crystals averages out the different absorption characteristics which can be rather significant as is well known by anybody old enough to have used a point detector on a four-circle goniometer.

Free electron laser X-rays (XFEL) and fast continuous read-out detectors have made their debut in the last three years. As the intensity of diffracted X-rays is proportional to the number of unit cells in the beam, this has made it possible to obtain data from crystals only a few µm in size, although the crystal is destroyed by the beam within a few femto seconds (serial femtosecond crystallography or SFX). Thus each image is a ‘still’ of a randomly orientated crystal. It is therefore not possible to use sequentially exposed images to reconstruct full reflections for scaling. Instead a ‘Monte Carlo’ procedure has been adopted by averaging hundreds of observations of the same reflection, each observation being subject to a different degree of partiality (Kirian *et al.*, 2010 ▶, 2011 ▶). Clearly this is an enormous waste and depends on the laws of chance. Alternative methods are currently being developed based on the post refinement procedures mentioned above (White *et al.*, 2012 ▶, 2013 ▶).

Synchrotrons started being used for X-ray crystallography in the early 1980s. Synchrotron data gave much cleaner patterns that usually extended further in resolution than what could be achieved from home X-ray sources. Initially, synchrotron radiation users were merely parasites on the physicists, but with time dedicated synchrotron sources were built, unencumbered by other priorities. The demand for dedicated synchrotron X-rays soon escalated resulting in the building of ever more powerful synchrotron sources. Although these new synchrotron sources do not compare with the intensity produced by XFEL radiation, nevertheless the latest generation of synchrotrons can also handle very small crystal sizes (Smith *et al.*, 2012 ▶). The paper by Gati *et al.* (2014 ▶) uses the PETRA III synchrotron at DESY in Hamburg to examine a frozen crystal slurry in which the average size of the needle-shaped crystals is about 11 µm long. These crystals were found *in vivo* in baculovirus-infected insect cells and therefore were not available for crystal growing optimization experiments. In the present case the authors were able to collect five or more consecutive frames for many of the tiny crystals in the slurry, using a rotation of 0.375°, before significant radiation destroyed the crystal. Hence they were able to determine full reflection intensities useful for scaling. On average each reflection was observed only about 12 times, far too few to permit the Monte Carlo technique. However, the paper is not explicit on exactly how scaling was achieved. The *R*_merge_ values of 0.71 overall and 2.69 for the outermost resolution shell at 3.0 Å are unduly high and presumably are the consequence of a different definition of *R*_merge_. Nevertheless, the structure was solvable by molecular replacement resulting in a structure that gave an *R*_work_ value of 0.223 and *R*_free_ of 0.264.

There is excellent agreement between the atomic positions determined for this structure when compared with an earlier 2.1 Å resolution structure determination of the same crystal form using XFEL radiation for crystals at room temperature delivered in a jet sprayed across the beam (Koopmann *et al.*, 2012 ▶). As presumably the point of the Gati *et al.* (2014 ▶) publication is to compare the synchrotron and XFEL data, it would have been worthwhile to give an *R* factor or correlation factor as a function of resolution between the structure amplitudes determined by the two procedures. A comparison between the structure amplitudes would be a direct comparison of the actual measurements made, whereas a comparison of the structures, as presented in the paper, means that the analysis of the observed structure amplitudes is mixed up with and drowned out by the additional information that goes into a structure determination. It would have been interesting to compare the size of errors for structure amplitudes of different magnitude, frame number, and how the error is dependent on frequency of observation, resolution, partiality, motion (in the current case) or position (in the SFX case) relative to the Ewald sphere. Consideration of the effects of radiation damage would also be of interest.
